# Digestion

**Published:** 1884-11

**Authors:** 


### HALL'S JOURNAL OF HEALTH

—AND—

### MEDICINE.

MONTHLY.

E. H. GIBBS, A. M., M. D., EDITOR.

FOUNDED IN 1854.

### DIGESTION.

A HEALTHY condition of the stomach is a guarantee against almost all diseases. The converse of this proposition is that a disordered stomach may be the cause of almost all diseases. Pure blood can no more be found in veins where the processes of digestion are defective than pure streams of water can flow from stagnant pools. A common error is to treat as diseases those manifestations that are only symptoms of trouble that exists at the fountain-head, which is the stomach. It is like cutting off the dead branches of a tree when the source of the disease is at the roots. Hence it is that ailments that have long resisted the action of medicines will often yield promptly to an improved condition of the digestive organs, brought about simply by the use of proper foods. And still it would be an extreme view to endorse the popular saying that food is better than medicine. Appropriate foods doubtless occupy the largest place in the list of remedies for disease, but medicines skillfully dispensed are often just as essential. The finest horse would be of no value, without the appliances necessary to harness him to his work. Medicines attach themselves to or attack diseases in some mysterious way, and the vital forces of nature are aroused and expel them from the system. Where disorder exists in the stomach without complication proper food alone may be sufficient to restore health; it may become both food and medicine.

Were it possible to always maintain the stomach in good working order, we should hardly know from experience what it is to be ill; but with the changes of climate and the vicissitudes inseparable from an active life occasional disorders of more or less gravity are unavoidable. As a rule, however, the remedy is at hand; we have only to know how to apply it, and to remember to act promptly and decidedly. When an engineer discovers that there is too much pressure in the boiler, he "hauls the fire"; he knows that if more steam is made than can be worked off by the engine, an explosion will be inevitable. So if the stomach is overladen, the organs which receive their supplies from the stomach become clogged and in time perhaps, permanently diseased. Thus persons who habitually eat too much, have besides the inevitable dyspepsia, either real disease of the heart, liver or kidneys, or else functional derangements of these organs. It is fortunate that in a great majority of cases patients become alarmed at these symptoms, and seek medical advice before there is actual disease. It should be understood that functional disturbance of the heart as indicated by palpitation almost always attends dyspepsia, and ceases when the dyspepsia is cured; but long neglected dyspepsia sometimes results in actual disease of the heart and kidneys. The most important injunction that can be given in reference to health, relates to eating and drinking. There can be no objection to the best and most nutritious foods that the market affords; the vital points are that they be properly cooked, and eaten slowly and in moderation. The worst form of dyspepsia is that caused by abstinence from nutritious food, since to the disorders that result from an insufficient diet is added debility, which renders recuperation difficult or impossible.

More suffering results from dyspepsia than from all other causes combined, and yet nine-tenths of it could be remedied by a little knowledge and a good deal of self-denial. An exclusive diet of "kumiss" and bread, for six months would cure any curable case of dyspepsia, besides the constipation, torpid liver, palpitation and other uncomfortable disorders that depend upon indigestion.

The August number of the Journal contained an article on "kumiss," but as many who read this article may not have seen that, we will briefly describe it and its uses. Dissolve a quarter of a compressed yeast cake in a quart of fresh milk,—about as warm as when first milked,—put it in a pitcher, stir occasionally, and in six or eight hours fermentation takes place; the milk thickens to about the consistence of buttermilk, and is then fit to use. Pour one-third of it into a bowl, with pieces of bread, add a little sugar, and eat as bread and milk. In the evening prepare another quart, using half a teacup of the "kumiss" instead of yeast. When once started the routine can go on indefinitely, just as in the making of buck-wheat cakes; using a little of the old kumiss to start the new. Kumiss contains all the elements of the best foods, and persons using it with bread exclusively for months, will require no other food, and will gain in flesh and in strength. Although not usually relished at first, those who use it for a time become very fond of it, and care little for other foods. The secret of the value of kumiss is in its fermentation. All other food that enters the stomach undergoes a process of fermentation, but when kumiss is eaten the stomach is spared the labor of this process in a great measure, and thus has an opportunity to rest and to recover from its irritable and congested condition, and the other organs also resume their natural condition.

